# High power density in a fully printed origami-inspired rolled thermoelectric generator enabled by paper self-folding

**DOI:** 10.1038/s41598-026-57024-0

**Published:** 2026-06-17

**Authors:** Hayato Yokoyama, Motoki Maeda, Giovanna Latronico, Paolo Mele, Hiroki Shigemune

**Affiliations:** 1 Graduate School of Engineering Science, Shibaura Institute of Technology, 3-7-5 Toyosu, Koto-Ku, Tokyo, 135-8548 Japan; 2 CNR-ICMATE, Institute of Condensed Matter Chemistry and Technologies for Energy, National Research Council, Via Previati 1/E, 23900 Lecco, Italy; 3 College of Engineering, Shibaura Institute of Technology, 3-7-5 Toyosu, Koto-Ku, Tokyo, 134-8548 Japan

**Keywords:** Thermoelectric generator, Printed electronics, Origami device, Self-folding, Paper-based device, Engineering, Materials science, Nanoscience and technology, Physics

## Abstract

Thermoelectric generators (TEGs) can harvest waste heat for small electronics. However, printed planar TEGs are often limited by low thermocouple density within a given footprint. Here, we present a paper-based rolled thermoelectric generator (Rolled TEG) that autonomously transforms from a planar sheet into a cylindrical geometry via paper self-folding using a fully printed fabrication process. Silver nanoparticle ink is inkjet printed to form electrodes and interconnects, while PEDOT:PSS is screen printed to form p-type thermoelectric legs, enabling series-connected architectures on paper substrates. The self-folding transformation rearranges thermocouples onto the inner cylindrical surface, increasing thermocouple density per projected installation area while preserving leg length and electrical connectivity. By systematically examining printing conditions and electrode pattern design, we clarify how internal resistance and output characteristics are governed and demonstrate that the rolled geometry enhances footprint-normalized power generation by achieving a footprint-normalized power density of 18.9 nW cm^−2^, which is 28.1 times higher than that of the Planar TEG. This work establishes autonomous paper self-folding as an effective design strategy for compact, fully printed thermoelectric devices.

**Supplementary Information:**

The online version contains supplementary material available at https://doi.org/10.1038/s41598-026-57024-0.

## Introduction

Thermoelectric generators, TEGs, are promising power sources for small electronics, including wearables, sensors, and IoT nodes^1–4^. TEGs generate a voltage from a temperature difference via the Seebeck effect. They have no moving parts, which enables maintenance-free and continuous energy harvesting. Conventional Bi_2_Te_3_-based thermoelectric modules show relatively high conversion efficiency^5–8^. However, they are brittle, costly, and dependent on scarce elements. Therefore, flexible TEGs using inexpensive and abundant materials have been actively studied^4,9–11^.

Printing technologies, such as screen printing and inkjet printing, can form conductive and thermoelectric inks on thin substrates^12–17^. They are attracting attention as routes to lightweight devices which can conform to curved surfaces^4^. However, flexible and printed TEGs commonly suffer from low power density. The active layers are thin, and printed materials often show lower thermoelectric performance than bulk materials. In addition, thermocouples are distributed on a planar footprint, which limits the thermocouple number per occupied area. As a result, the generated power per unit area tends to be small. Distributed sensors and microelectronic circuits require both high power density and a small footprint. Yet many printed TEGs still use only a few to a few tens of thermocouples^18,19^. This indicates that improving thermocouple density remains an important challenge.

As an effective strategy, three-dimensional architectures have been proposed to increase thermocouple density within a limited footprint^20–22^. These methods transform planar patterns into origami-like structures or rolled structures. They stack thermocouples densely while preserving leg length and electrical interconnections. Recently, rolled organic TEGs have also been reported by winding thermoelectric legs on ultrathin substrates^23^. Such approaches geometrically expand the thermocouple number and the effective temperature gradient, which are constrained in planar designs. They are therefore positioned as routes to secure the power density required for small devices^4^. Although recent rolled, origami-like, and pop-up TEGs also transform planar layouts into three-dimensional architectures, the present work differs in its design objective and formation process. The Rolled TEG in this study aims to improve footprint-normalized power density by converting a printed Planar TEG into a compact rolled geometry within a limited projected footprint, whereas many pop-up TEGs are designed to control heat paths or effective temperature differences. In addition, the rolled geometry is formed autonomously by paper self-folding through solution application alone, without external jigs, actuators, or mechanical manipulation.

However, fabrication processes to obtain three-dimensional structures often require additional jigs or actuators^24,25^. From the viewpoints of simplicity and scalability, these processes still have room for improvement. Paper self-folding exploits the interaction between paper and a solution to induce spontaneous bending or rolling along preprinted patterns^26,27^. The shape change is triggered by solution application alone, without external mechanical energy. Paper self-folding therefore offers shape programmability, reproducibility, and low energy actuation^28–30^. Paper is also an attractive, flexible substrate candidate. It is low-cost, lightweight, and environmentally benign, and it is compatible with printing processes such as inkjet printing. Self-folding paper devices, including highly stretchable sensors and paper robots, have been reported by combining paper self-folding with printing^26,28–30^. In parallel, many printed TEGs using paper substrates without self-folding have also been reported^6,18,19^. However, the concept remains underexplored for organic flexible devices where all constituents are organic materials. This includes the substrate, electrodes, and thermoelectric materials, and it targets both high thermocouple density and power density.

In this study, we propose a paper-based rolled thermoelectric generator (TEG) that integrates autonomous paper self-folding with a fully printed fabrication process. By combining inkjet-printed silver nanoparticle electrodes with screen-printed PEDOT:PSS thermoelectric legs on a paper substrate, a planar device is converted into a cylindrical architecture without external mechanical actuation. This self-folding transformation converts the printed Planar TEG into a compact rolled geometry while preserving the original electrode layout, length, and electrical series connections. This geometry improves footprint-normalized power generation within a limited projected footprint. Through a direct comparison between planar and rolled configurations, we demonstrate that the rolled architecture substantially enhances footprint-normalized power density mainly by reducing the projected footprint rather than by substantially increasing the absolute maximum output power, whereas internal resistance and open-circuit voltage remain largely unchanged after rolling, indicating robust electrical integrity under strong bending. By systematically examining geometric and printing-related factors, we further identify design conditions that balance low internal resistance with high output performance. These results establish autonomous paper self-folding as an effective strategy for improving device-level power density in fully printed organic TEGs without increasing planar area, offering a scalable platform for distributed and environmentally friendly IoT applications, including wearable and ambient sensing systems^4,11,31–34^.

## Results and discussion

### Rolled TEG concept and fabrication

**Fig. 1:**
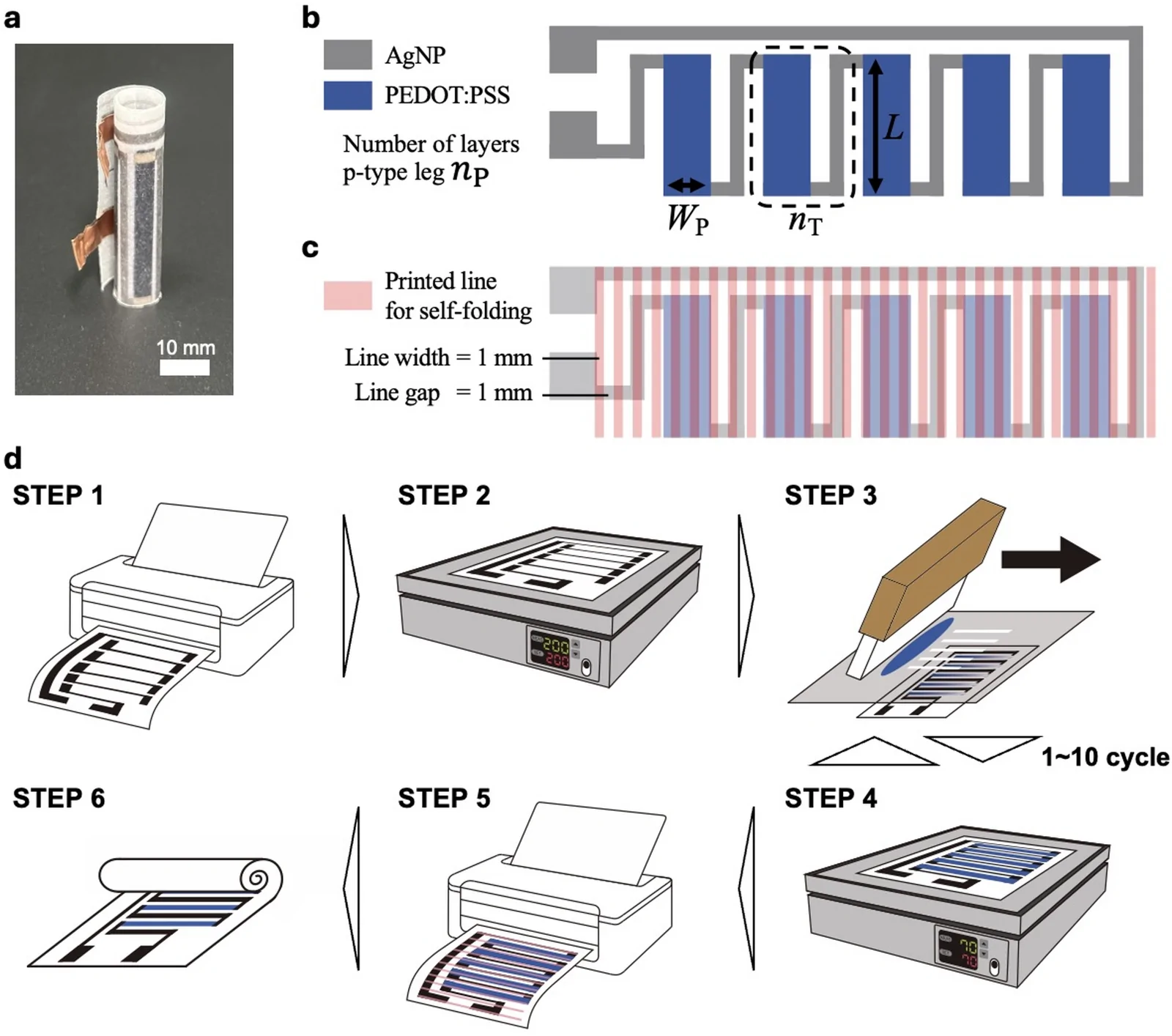
Device concept and fabrication process. (**a**) Photograph of the Rolled TEG, which autonomously transforms from a planar geometry into a rolled geometry via paper self-folding. (**b**) Layout of the electrodes and thermoelectric legs with pi-shaped interconnects. The interconnects are concentrated on the upper side of the TEG, preventing obstruction of heat flow into the thermoelectric legs and minimizing thermal shunts. (**c**) Printed line pattern for paper self-folding. Fine, parallel fold lines are arranged at a constant pitch on the paper substrate to induce unidirectional folding and cylindrical rolling. (**d**) Fully printed fabrication flow. Step 1, inkjet printing of AgNP interconnects on a paper substrate. Step 2, thermal treatment of the AgNP interconnects by drying and sintering. Step 3, screen printing of PEDOT:PSS legs. Step 4, drying of the PEDOT:PSS legs; Steps 3 and 4 are repeated depending on *n*_P_. Step 5, activation of paper self-folding along the predefined fold-line pattern. Step 6, self-folding of the device.

In this study, we propose a method that converts a planar, fully printed TEG into a rolled geometry by combining a paper substrate with paper self-folding. This approach packs thermocouples densely within a limited footprint and enables a rolled TEG designed for high footprint-normalized power density. Key geometric and printing-related parameters, including leg dimensions, thermocouple density, and film thickness, are systematically investigated in this work. Figure 1a shows a rolled, origami-inspired thermoelectric device formed by paper self-folding. In the planar state, p-type thermoelectric legs made of PEDOT:PSS and conductive interconnects formed from silver nanoparticle ink are fully printed on the same surface. The pattern electrically connects each thermocouple in series and is designed to keep both terminals accessible after rolling. After printing and activation of the self-folding mechanism, the substrate autonomously forms a cylindrical structure, increasing power density while preserving the original leg length defined in the planar design.

Figure 1b shows the electrode pattern adopted in this study. The pattern employs a pi-shaped architecture, where adjacent legs are alternately connected to the hot side and the cold side. Each leg generates an electromotive force via the Seebeck effect, and the wiring reliably adds these voltages in series. This design avoids current cancellation between legs and prevents voltage dispersion caused by unintended parallel paths. Accordingly, the open-circuit voltage, *V*_OC_, increases approximately in proportion to the thermocouple number, *n*_T_, which enhances the maximum output under load-matching conditions. In addition, the interconnects are concentrated on the upper side of the TEG, preventing obstruction of heat flow into the thermoelectric legs and minimizing thermal shunts. As illustrated in Fig. 1b, the key geometric and printing-related design parameters are the p-type leg width, *W*_P_, the leg length, *L*, the thermocouple number, *n*_T_, and the number of printed PEDOT:PSS layers, *n*_P_.

Figure 1c illustrates the origami-based folding pattern used for paper self-folding. Fine, parallel fold lines are introduced at a constant pitch on the paper substrate, along which the paper preferentially folds. When the fold lines are placed at a small and uniform spacing, a continuous stress gradient is formed across the patterned region, resulting in a cylindrical roll. In this study, the fold line width and spacing were both set to 1 mm, which enables reproducible transformation into a rolled geometry. Both the AgNP interconnects and the PEDOT:PSS p-type legs are fabricated as thin films on the paper substrate. Because of their low bending stiffness, these thin-film electrodes and thermoelectric legs can accommodate large curvature and are folded together with the paper along the printed pattern. In addition, the folding solution can be applied over the functional layers, and self-folding remains effective after printing the electrodes and thermoelectric legs. As a result, a tightly rolled cylindrical geometry is formed while preserving electrical continuity.

Figure 1d outlines the fabrication process. We used a fully printed workflow that combines inkjet printing and screen printing. First, we inkjet print AgNP ink on the paper substrate to form interconnects, then we sinter them on a 200 °C hot plate in air. Next, we screen print PEDOT:PSS as the *p* type material and dry it for 10 min on a 70 °C hot plate in air. We repeat the PEDOT:PSS printing and drying steps to set *n*_P_. For the Planar TEG, the process ends after forming the legs and interconnects. Finally, self-folding of the device is triggered via inkjet printing onto predefined regions, completing the Rolled TEG. The printing procedure based on paper self-folding was based on our previous studies^26,27^. The present device uses p-type PEDOT:PSS legs and AgNP interconnects, and therefore does not form a true p–n thermocouple configuration. Because the Seebeck coefficient of Ag is close to zero, the effective Seebeck coefficient of each thermocouple is mainly governed by the p-type PEDOT:PSS leg. Printed TEGs using only p-type thermoelectric materials with metal interconnects have been reported in previous studies, and these devices can generate *V*_OC_ in response to a temperature difference^10,13,17^. However, this is a limitation of the current device architecture compared with p–n thermocouples using both p-type and n-type thermoelectric materials. Incorporating printed n-type thermoelectric materials, such as inks based on Ag_2_Se, n-type polymer inks, or carbon-based n-type inks, would be a promising route to increase the effective Seebeck coefficient. However, printed n-type materials may also introduce additional internal resistance, contact resistance, and process compatibility issues. Therefore, even if the effective Seebeck coefficient is increased by introducing n-type materials, the overall output power may not necessarily improve unless the increase in resistance is suppressed. Future integration of n-type materials requires careful optimization of material conductivity, printability, interfacial contact, and compatibility with the paper self-folding process.

### Measurement setup and evaluation metrics

**Fig. 2:**
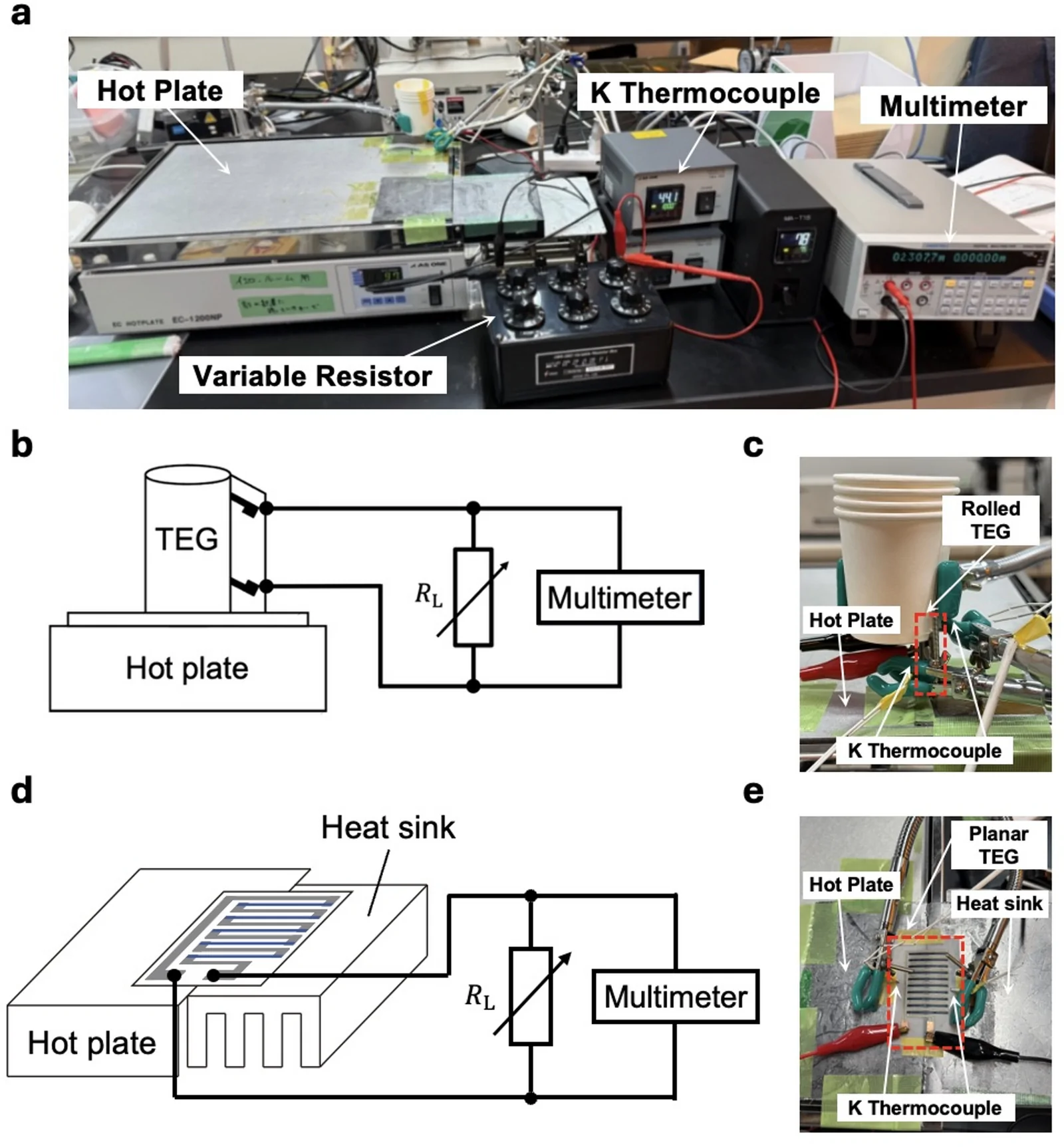
Measurement setup and circuit configuration. (**a**) Photograph of the measurement system. A hot plate is used as the heat source, and the load voltage, *V*_L_, is measured through an external load resistance, *R*_L_. (**b**) Circuit diagram for the Rolled TEG measurement. The Rolled TEG is connected to *R*_L_, and *V*_L_ is recorded while sweeping *R*_L_. (**c**) Photograph of the Rolled TEG during measurement. The cylindrical device is placed upright on the hot plate, and Δ*T* is applied between the upper and lower ends of the device. (**d**) Circuit diagram for the Planar TEG measurement. (**e**) Photograph of the Planar TEG during measurement. The device is placed between a hot plate and an aluminum heat sink, applying Δ*T* along the leg direction.

In this section, we compared the power generation characteristics of the Planar TEG and the Rolled TEG before rolling, then we quantitatively evaluated how the shape change affects output performance. For this purpose, we built a measurement system that can control the temperature difference, Δ*T*, and we obtained the output voltage, current, and power density. Figure 2a shows an overview of the measurement setup constructed in this study. For both the Planar TEG and the Rolled TEG, a hot plate was used as the heat source to apply Δ*T* to the device, and the load voltage, *V*_L_, was measured through an external load resistance, *R*_L_, using a variable resistor.

Figure 2b shows the electrical measurement circuit used for the Rolled TEG, where the load voltage across an external resistance *R*_L_, is measured. Figure 2c shows a photograph of the Rolled TEG during measurement. In Fig. 2c, we used a fixture that covered the top of the Rolled TEG with a paper cup to stably fix the device at a predetermined position. The Rolled TEG was placed upright on the hot plate, and the paper cup was placed over it to hold the device. We placed weights inside the paper cup to apply a moderate load to the Rolled TEG, which maintained its posture and contact condition, reduced variation between samples and between measurement runs, and ensured measurement reproducibility. For the Rolled TEG, Δ*T* was defined as the difference between the temperatures measured near the lower end and near the upper end on the side surface, using K-type thermocouples. The Rolled TEG configuration prioritizes compactness and installation efficiency rather than optimal thermal boundary conditions, and the present setup therefore represents a conservative estimate of its achievable power.

Figure 2d shows the electrical measurement circuit used for the Planar TEG, where the load voltage across an external resistance is measured under a defined temperature difference. Figure 2e shows a photograph of the Planar TEG during measurement. For the Planar TEG, the device was placed across a hot plate and an aluminum heat sink, and Δ*T* was applied along the leg direction by bringing the paper substrate at the leg and locations into contact with the heat sink. For the Planar TEG, Δ*T* was determined as the difference between the temperatures measured by K-type thermocouples attached to the paper substrate surface at the positions corresponding to the leg ends on the hot plate side and on the heat sink side, rather than by attaching thermocouples to the leg ends themselves. The heat sink functioned as the cold side, which provided a stable temperature gradient against the hot plate.

In each measurement, *V*_L_ was recorded while sweeping *R*_L_, and the output power, *P*, was calculated from the measured values using the following equation.

1 $$P=\frac{{{V}_{\mathrm{L}}}^{2}}{{R}_{\mathrm{L}}}$$

In addition, the maximum power density, *P*_max,d_, was defined as the maximum power per unit area, obtained from the power–voltage (P–V) curve, as given by the following equation.

2 $${P}_{\mathrm{m}\mathrm{a}\mathrm{x},\mathrm{d}}=\frac{{P}_{\mathrm{m}\mathrm{a}\mathrm{x}}}{{S}_{\mathrm{T}\mathrm{E}\mathrm{G}}}$$

Here, *S*_TEG_ denotes the effective footprint required to install the device, which corresponds to the total area of the unfolded sheet for the Planar TEG and the projected top-view area when the Rolled TEG stands upright. *S*_TEG_ does not represent the effective power generation area, but rather the physical installation area occupied by the device. In this study, we focus on device-level output characteristics and footprint-normalized power generation rather than thermoelectric conversion efficiency. The thermal resistance of the Planar and Rolled TEGs was not measured because accurate evaluation requires consideration of heat transfer through the paper substrate, PEDOT:PSS films, AgNP interconnects, contact interfaces, and air gaps inside the rolled structure. In addition, the Planar and Rolled TEGs have different thermal boundary conditions, making direct efficiency comparison nontrivial. Therefore, thermal-resistance-based ZT estimation and conversion-efficiency analysis will be addressed in future work.

### Effect of electrode and p-leg geometries on device performance

In this section, we aim to derive practical design guidelines for electrode and leg patterns that improve the electrical output characteristics of the Rolled TEG. We systematically analyze how geometric and process factors affect the internal resistance, *R*_i_, the open circuit voltage, *V*_OC_, the maximum power, *P*_max_, and the maximum power density, *P*_max,d_. The factors considered are the leg length, *L*, the p-type leg width, *W*_P_, the thermocouple number, *n*_T_, and the number of printed PEDOT:PSS layers, *n*_P_. Based on these results, we summarize practical guidelines for geometry and printing conditions.

**Fig. 3:**
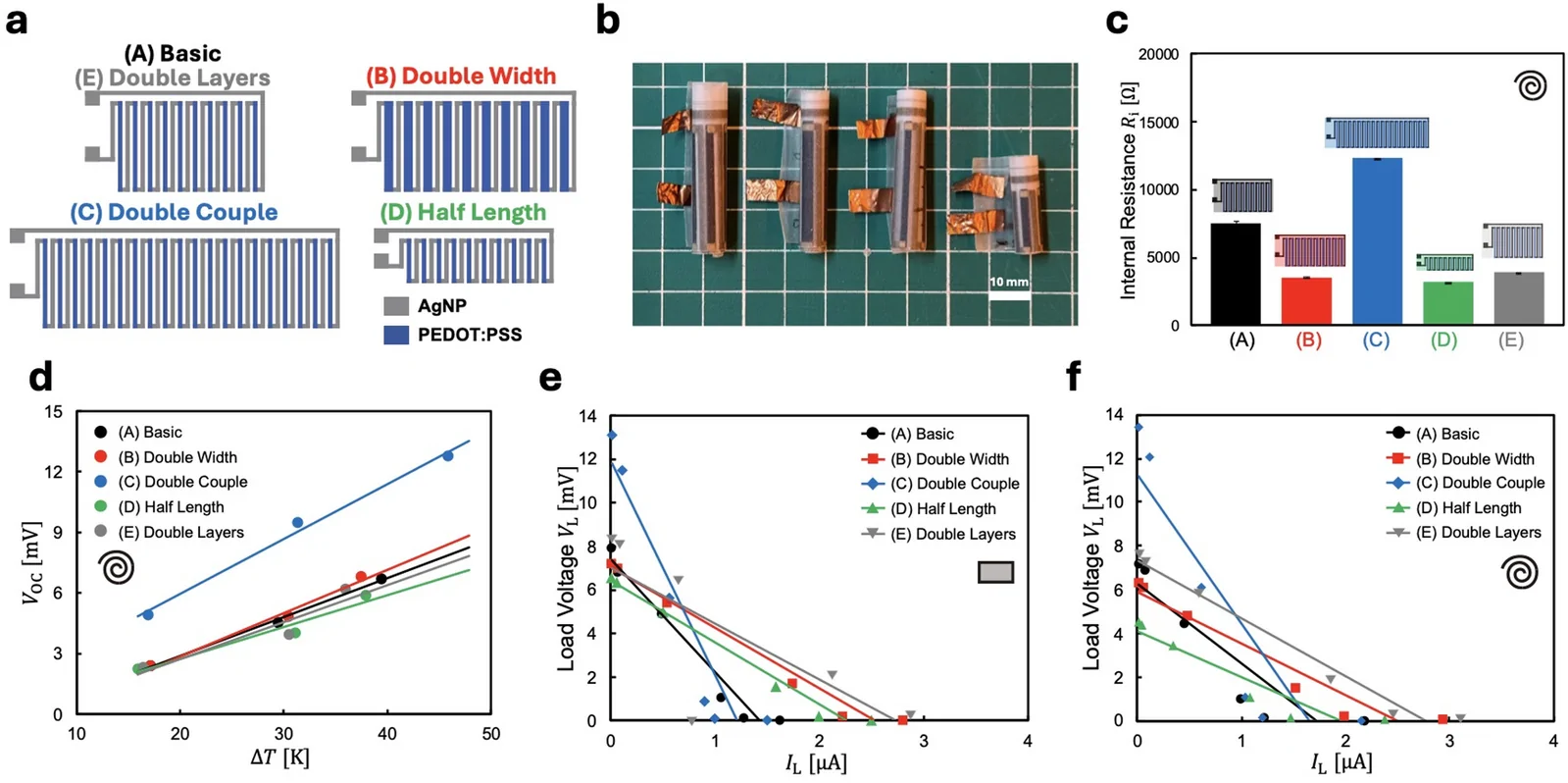
Effects of electrode and leg geometries on *R*_i_, *V*_OC_, and *I*_L_–*V*_L_. (**a**) Leg designs of the fabricated TEGs. Five patterns are compared, namely Basic, Double Width, Double Couple, Half Length, and Double Layers. (**b**) Photographs of the Rolled TEGs after self-folding. All patterns are transformed into a rolled geometry by paper self-folding. (**c**) Measured *R*_i_ of the Rolled TEGs. Compared with Basic, *R*_i_ decreases for Double Width and Double Layers, and it further decreases for Half Length. In contrast, *R*_i_ increases for Double Couple. (**d**) Dependence of *V*_OC_ on Δ*T*. *V*_OC_ shows a clear linear relationship with Δ*T* for all devices, and Double Couple exhibits an twofold higher *V*_OC_ than Basic, consistent with its doubled thermocouple number. (**e**) *I*_L_–*V*_L_ characteristics of the Planar TEGs at a hot plate temperature of 100 °C. All designs show linear *I*_L_ to *V*_L_ relations, and differences in *R*_i_ appear as changes in slope. (**f**) *I*_L_–*V*_L_ characteristics of the Rolled TEGs at 100 °C*.* A similar linear voltage response is maintained after rolling, indicating that the electrical characteristics are preserved through the self-folding process.

Figure 3a shows the electrode patterns and p-type leg printing patterns used in this study, and Fig. 3b shows photographs of the fabricated Rolled TEGs. Table 1 summarizes the detailed dimensions and parameter sets for each design. Taking design (A), Basic, as the reference, we prepared four additional designs, (B) Double Width with doubled *W*_P_, (C) Double Couple with doubled* n*_T_, (D) Half Length with halved *L*, and (E) Double Layers with *n*_P_ increased to 10. Their electrical characteristics were compared under identical measurement conditions. As shown in Fig. 3b, all Rolled TEGs converged to nearly the same cylindrical size after self-folding, indicating that the self-folding process is robust against variations in electrode and leg geometries.

**Table 1:** Geometric and printing parameters of the devices.

Type	Electrode length *L* [mm]	p-type electrode width *W*_p_ [mm]	Number of thermocouple* n*_T_	Number of printed PEDOT:PSS layers* n*_P_
Basic	30	1.5	10	5
Double width	30	3.0	10	5
Double couple	30	1.5	20	5
Half length	15	1.5	10	5
Double layers	30	1.5	10	10

Figure 3c to f show the measurement results of the fabricated devices. The spiral and rectangular symbols in Fig. 3c to f indicate whether the device was measured in the Rolled or Planar geometry. Figure 3c compares the internal resistance, *R*_i_, among the Rolled TEG designs, where clear trends are observed across the design factors. Relative to (A) Basic with *R*_i_ = 7.48 × 10^3^ Ω, Double Width and Half Length show reduced resistance because *W*_P_ is increased and *L* is halved. For example, Half Length shows *R*_i_ = 3.18 × 10^3^ Ω, which corresponds to a large 57.5% decrease compared with Basic. In contrast, (C) Double Couple shows *R*_i_ = 1.23 × 10^4^ Ω, which is a 64.6% increase compared with Basic. By comparison, (E) Double Layers shows* R*_i_ = 3.91 × 10^3^ Ω, which corresponds to a 47.7% decrease. Overall, increasing *W*_P_ or decreasing *L* reduces the resistance of each leg by widening or shortening the conduction path, whereas increasing *n*_T_ increases *R*_i_ due to the series addition of leg and interconnect resistances. These trends are consistent with previous reports on geometric optimization of thermoelectric modules and printed PEDOT:PSS-based flexible TEGs, where reduced sheet resistance with increased film thickness and increased internal resistance with higher thermocouple numbers have been reported^35–37^.

Figure 3d shows the dependence of the open-circuit voltage, *V*_OC_, on the temperature difference, Δ*T*, for each design. *V*_OC_ increases linearly with Δ*T* for all devices, consistent with the Seebeck effect. Except for Double Couple, all designs exhibit nearly identical *V*_OC_ values over the entire Δ*T* range. This behavior reflects the fact that Double Couple has a doubled thermocouple number, *n*_T_ = 20, compared with *n*_T_ = 10 for Basic. For example, at Δ*T* = 16.9 K, Double Couple shows *V*_OC_ = 4.90 mV, whereas Basic shows *V*_OC_ = 2.40 mV at a comparable Δ*T* = 17.2 K, corresponding to an approximately twofold increase. At higher Δ*T*, this voltage enhancement is preserved, indicating that the proportionality between *V*_OC_ and *n*_T_ is maintained over the measured temperature range. In contrast, variations in *W*_P_ or *n*_P_ have little influence on *V*_OC_, indicating that these parameters primarily affect the internal resistance rather than the generated voltage.

When comparing the Planar TEG and the Rolled TEG, *V*_OC_ for the Rolled TEG tends to be slightly lower in the same Δ*T* range. However, the slopes of the Δ*T*-*V*_OC_ characteristics are nearly identical for both geometries, indicating that the intrinsic voltage sensitivity is largely preserved after transformation from the planar to the rolled configuration. This slight reduction in *V*_OC_ can be attributed to differences in the thermal boundary conditions between the two measurement setups. In the Planar TEG, both ends of the thermoelectric legs are in direct contact with the hot plate and the aluminum heat sink, establishing a well-defined temperature difference along the leg direction. In contrast, in the Rolled TEG, only a narrow paper rim near the lower end contacts the hot plate, while the upper end is not attached to a heat sink and is primarily cooled by heat exchange with the surrounding air. As a result, although Δ*T* is evaluated from the temperature difference between the lower and upper ends of the cylinder, the effective temperature difference applied to each thermocouple is expected to be smaller than that in the Planar TEG.

Figure 3e shows the relationship between the load current, *I*_L_, and the load voltage, *V*_L_, for the Planar TEG, and Fig. 3f shows the corresponding relationship for the Rolled TEG. In both geometries, *V*_L_ decreases linearly with increasing *I*_L_, and the measured data for each design can be well approximated by a straight line. The intercept corresponds to *V*_OC_, while the slope represents *R*_i_, allowing differences in operating points and resistance to be distinguished among the designs. For the Rolled TEG, the same linear relationship is preserved, and the maximum power is obtained near the operating point where the external load resistance is matched to *R*_i_.

### Effect of PEDOT:PSS layer number on electrical and power performance

**Fig. 4:**
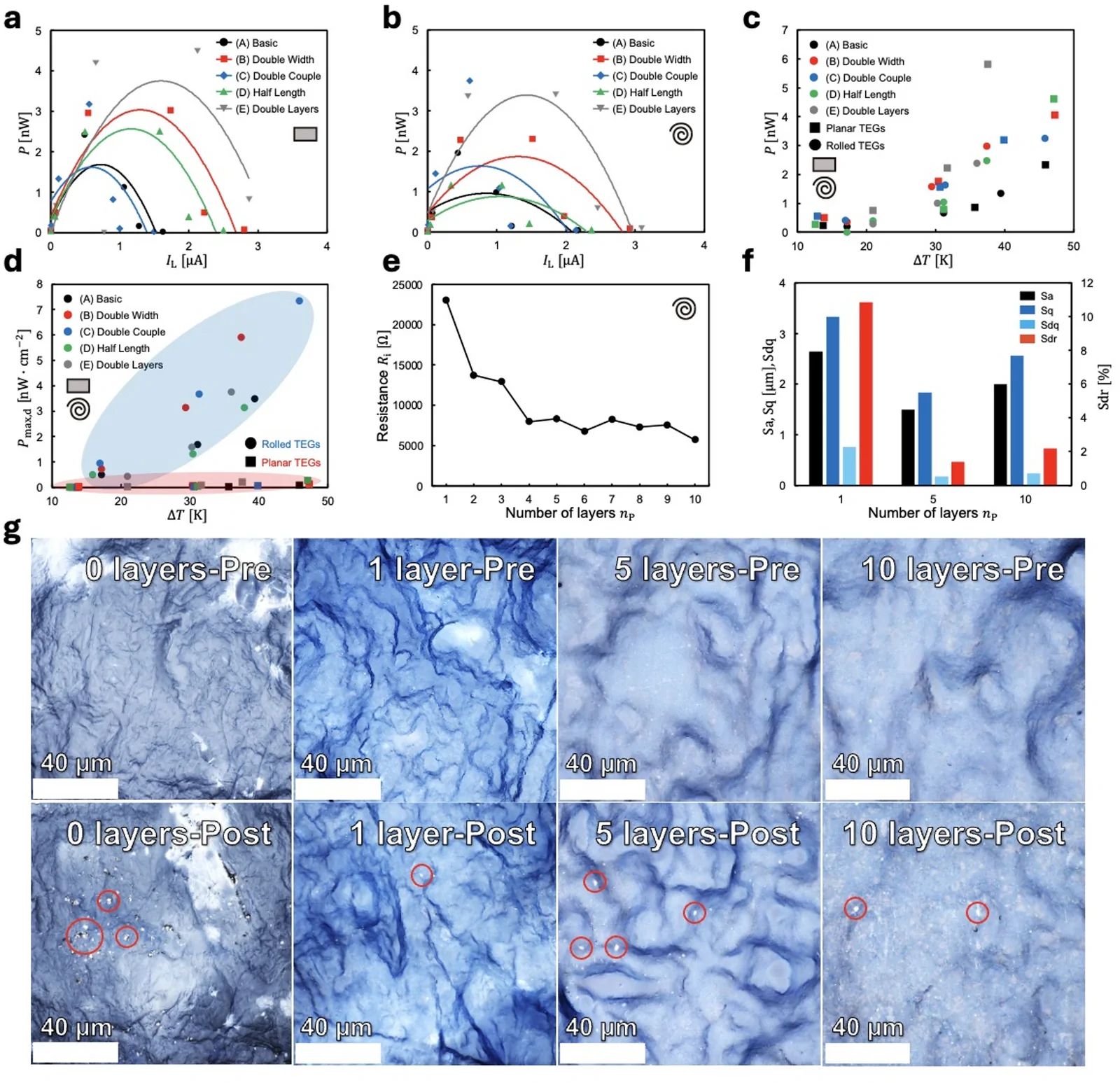
Output characteristics of the Planar and Rolled TEGs and the effect of PEDOT:PSS layer number. (**a**) *P* as a function of *I*_L_ for the Planar TEG at a hot plate temperature of 100 °C. (**b**) *P*–*I*_L_ characteristics of the Rolled TEG at 100 °C, showing a shift of the peak toward lower current and lower power after rolling. (**c**) *P*_max_ as a function of Δ*T*, for each design, where the Planar TEG generally exhibits higher *P*_max_. (**d**) *P*_max,d_ showing a pronounced enhancement in the Rolled geometry. (**e**) Dependence of *R*_i_ on *n*_P_, showing a rapid decrease in the few-layer regime and saturation at higher *n*_P_. (**f**) *n*_P_ dependence of surface roughness parameters (*S*_*a*_, *S*_*q*_, *S*_*dq*_, and *S*_*dr*_) of the PEDOT:PSS legs, indicating surface planarization with increasing *n*_P_. (**g**) Laser microscope images of the paper substrate and PEDOT:PSS films before (Pre) and after (Post) self-folding, illustrating film coverage, surface smoothing, and local defects induced by rolling.

In this section, we focus on the role of the PEDOT:PSS layer number, *n*_P_, in determining the internal resistance and output characteristics of the Rolled TEG. Figure 4a and b show the power–current (*P–I*) characteristics of the Planar TEG and the Rolled TEG measured at a hot plate set temperature of 100 °C. In both geometries, the current–voltage (*I–V*) characteristics are highly linear, resulting in parabolic *P–I* curves with a well-defined maximum power, *P*_max_, at the vertex. The overall shapes of the *P–I* curves are similar for the Planar and Rolled geometries, indicating that the fundamental electrical behavior is preserved after rolling. In the Rolled TEG, however, the vertex of the *P–I* curve shifts toward lower current for many designs, and the entire curve tends to shift toward lower power. This behavior suggests that rolling alters the operating point at which *P*_max_ is achieved, leading to a reduction in output power.

Figure 4c compares the maximum power, *P*_max_, of the Planar TEG and the Rolled TEG under temperature difference, Δ*T*, conditions corresponding to hot plate set temperatures of 50, 75, and 100 °C. For design (C) Double Couple, the measured Δ*T* for the Planar TEG is several kelvin smaller than that for the Rolled TEG, which is attributed to differences in thermal resistance paths and thermocouple positions between the two measurement setups. The Planar TEG generally exhibits higher *P*_max_ values. For the Rolled TEGs, the maximum power density, *P*_max,d_, increases monotonically with increasing Δ*T* across all designs. The separation between the Planar and Rolled geometries is more pronounced than the differences among individual design variations, indicating that geometric transformation has a larger impact on footprint-normalized performance within the examined range. The absolute maximum output power, *P*_max_, of the Rolled TEG was not substantially higher than that of the corresponding Planar TEG. This indicates that the main advantage of the Rolled TEG is not an increase in *P*_max_ itself, but the improvement in footprint-normalized maximum power density, *P*_max,d_, achieved by compactly rearranging the printed thermocouple array within a smaller projected footprint.

As an example, design (E) Double Layers exhibits a high *P*_max_ because multilayer PEDOT:PSS legs reduce the leg resistance and keep* R*_i_ low, while *V*_OC_ remains comparable to other designs. In contrast, design (C) Double Couple increases *V*_OC_ by doubling the thermocouple number, however *R*_i_ also increases due to the series addition of leg and interconnect resistances, resulting in *P*_max_ values comparable to those of Double Layers. After transformation from the Planar to the Rolled geometry, Δ*T* tends to decrease and the operating point shifts toward lower current, indicating an increase in *R*_i_. As a result, *P*_max_ of the Rolled TEG generally becomes lower than that of the Planar TEG. Among the designs examined, Double Layers shows the highest *P*_max_ in the Planar geometry, whereas Double Couple exhibits the highest *P*_max_ among the Rolled TEG designs owing to its higher *V*_OC_.

Figure 4d compares the maximum power density, *P*_max,d_ between the Planar and Rolled geometries. Although Fig. 4c shows that the Planar TEG exhibits higher *P*_max_, *P*_max,d_ is consistently higher for the Rolled TEG under the same Δ*T* conditions. For example, for design (C) Double Couple at a hot plate set temperature of 100 °C, *P*_max,d_ increases from 0.0649 nW cm^−2^ in the Planar geometry to 7.35 nW cm^−2^ in the Rolled geometry, corresponding to a 112-fold increase. Across all examined conditions, the Rolled TEG exhibits higher *P*_max,d_ than the Planar TEG by factors ranging from 11-fold, for 100 °C and Half Length, to 117-fold, for 75 °C and Double Couple. This enhancement arises from the geometric transformation by paper self-folding, which preserves the leg length and thermocouple number while substantially reducing the projected installation footprint.

Figure 4e shows the dependence of the internal resistance, *R*_i_, of the Rolled TEG on the number of printed PEDOT:PSS layers, *n*_P_. At *n*_P_ = 1, *R*_i_ is high, and it decreases rapidly as *n*_P_ increases to a few layers. At intermediate layer numbers, *R*_i_ decreases to the order of several kΩ and then shows only a gradual decrease, approaching saturation at higher *n*_P_. At *n*_P_ = 10, Ri reaches its lowest value in the examined range. Based on this trend, *n*_P_ = 10 was selected for subsequent experiments to achieve lower internal resistance and improved output performance.

To examine whether the reduction in *R*_i_ is associated with surface changes induced by increasing *n*_P_, Fig. 4f evaluates the surface roughness of the PEDOT:PSS legs based on height data acquired by laser microscopy. Figure 4f shows the dependence of several roughness metrics on *n*_P_. The arithmetic mean height, *S*_*a*_, decreases markedly from the single-layer condition to a few-layer regime and then shows only a modest change at higher *n*_P_. Similar trends are observed for the root mean square height, *S*_*q*_, the root mean square gradient, *S*_*dq*_, and the developed interfacial area ratio, *S*_*dr*_, particularly in the low-*n*_P_ regime. At low *n*_P_, the PEDOT:PSS surface is relatively rough, characterized by large local slopes and an increased developed surface area. With increasing *n*_P_, valleys are progressively filled and the film becomes smoother and more continuous. At higher *n*_P_, the surface largely maintains this flattened morphology with only minor variations.

Figure 4g shows laser microscope images of the paper substrate and PEDOT:PSS film surfaces. The top row labeled “Pre” shows the surfaces before self-folding, and the bottom row labeled “Post” shows the surfaces after self-folding. The columns correspond to the paper substrate and PEDOT:PSS films with *n*_P_ = 1, 5, and 10. As *n*_P_ increases, the PEDOT:PSS film progressively covers the paper substrate, and the influence of the underlying paper fiber topography becomes less pronounced. At *n*_P_ = 1, paper fiber structures remain visible, whereas at *n*_P_ = 5, valleys are largely filled and a more continuous film is formed. After self-folding, localized bright regions are observed on the PEDOT:PSS surfaces, particularly at higher *n*_P_. These features appear as micropores or localized delamination and are likely associated with local stress induced during roll formation. Such micro defects do not dominate the overall roughness trend discussed in Fig. 4f but may represent a secondary effect at higher layer numbers. Taken together, these observations indicate that *n*_P_ affects device performance not only through the deposited PEDOT:PSS amount but also through process-induced film morphology. Increasing *n*_P_ improves PEDOT:PSS film coverage and continuous conduction paths on the paper substrate, contributing to the decrease in *R*_i_, whereas localized defects formed during rolling may partly contribute to the increase in *R*_i_ and are consistent with the decrease in *P*_max_ after rolling. As an initial assessment of long-term electrical stability after rolling, *V*_OC_ and *R*_i_ were monitored over 14 days, as shown in Supplementary Figs. S2 and S3. The Rolled TEG maintained stable *V*_OC_ at hot plate set temperatures of 50, 75, and 100 °C, and *R*_i_ showed only a small change during the measurement period. These results indicate that the basic thermoelectric response and electrical continuity were preserved over the examined period, despite the local defects observed after rolling. The present long-term evaluation was limited to electrical stability after rolling, and repeated mechanical deformation under bending or pressing was not systematically investigated. Quantitative evaluation of such mechanical cycling effects would require dedicated mechanical reliability tests.

### Effect of p-type leg width on output performance

**Fig. 5:**
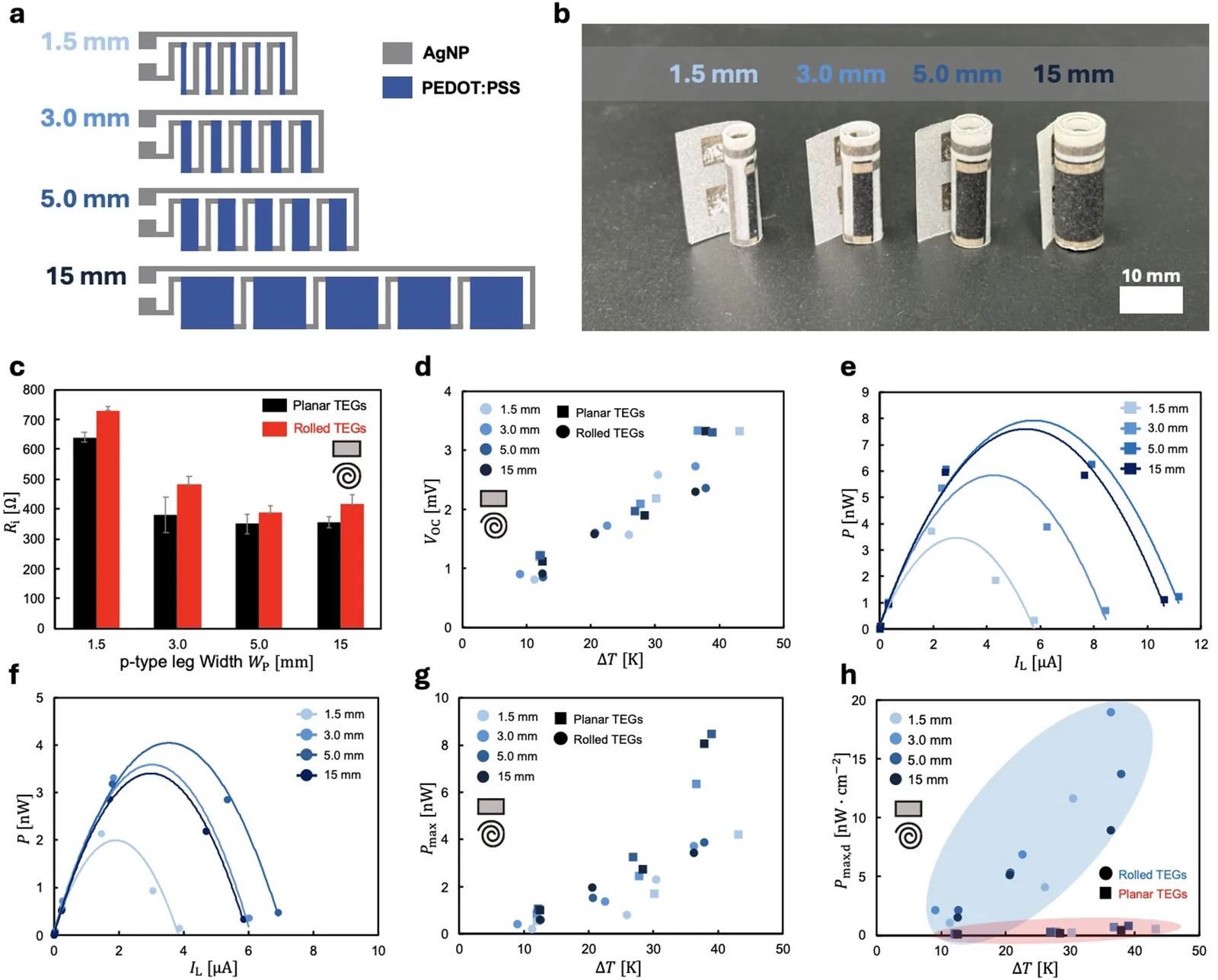
Structure and power generation characteristics with different *W*_P_. (**a**) Pattern designs with *W*_P_ = 1.5, 3.0, 5.0, and 15 mm. (**b**) Photographs of the Rolled TEGs after self-folding. (**c**) *R*_i_ as a function of *W*_P_ for both geometries, showing a decrease with increasing *W*_P_ and saturation at *W*_P_ = 5.0 mm. (**d**) Δ*T* dependence of *V*_OC_. All devices show clear linearity, and the slope remains unchanged with varying *W*_P_. (**e**) *P*-*I*_L_ characteristics of the Planar TEGs at 100 °C. The largest vertices are observed at *W*_P_ = 5.0 mm. (**f**) *P*-*I*_L_ characteristics of the Rolled TEGs at 100 °C. The output reaches its maximum at *W*_P_ = 5.0 mm, as observed for the planar TEG. (**g**) Δ*T* dependence of *P*_max_. The output is higher for the Planar TEG than for the Rolled TEG, while within each geometry, *W*_P_ = 5.0 mm provides the highest *P*_max_. (**h**) Δ*T* dependence of *P*_max,d_. The Rolled TEGs exhibit higher *P*_max,d_ than the Planar TEGs, with the largest enhancement observed at *W*_P_ = 3.0 mm.

In this section, we investigate whether increasing the p-type leg width, *W*_P_, is an effective strategy for improving output performance, using both Planar and Rolled TEGs with systematically varied leg widths. In the previous section, we showed that increasing leg width reduces internal resistance and improves output. Here, *W*_P_ is varied from *W*_P_ = 1.5 to 15 mm while keeping *L*, *n*_T_, and *n*_P_ constant. Figure 5a shows the pattern designs of the Planar TEGs with *W*_P_ = 1.5, 3.0, 5.0, and 15 mm. Table 2 summarizes the specific design parameters.

**Table 2:** Design parameters for p-type leg width variation.

Type	Electrode length *L* [mm]	p-type electrode width *W*_p_ [mm]	Number of thermocouple *n*_T_	Number of printed PEDOT:PSS layers *n*_P_
1.5	15	1.5	5	10
3.0	15	3.0	5	10
5.0	15	5.0	5	10
15	15	15	5	10

Figure 5b shows photographs of the Rolled TEGs obtained by applying self-folding. For all *W*_P_ values, the paper substrate autonomously rolls into a stable cylindrical structure, confirming that the thermoelectric legs are consistently arranged along the inner sidewall of the Rolled TEG. Figure 5c shows the dependence of the internal resistance, *R*_i_, on *W*_P_ for both the Planar and Rolled TEGs. In the Rolled TEG, *R*_i_ decreases markedly as *W*_P_ is increased from 1.5 to 5.0 mm, whereas further widening to 15 mm results in little additional reduction and a slight increase in *R*_i_, indicating saturation of the leg-width effect around *W*_P_ = 5.0 mm. This saturation suggests that the resistance of the PEDOT:PSS legs becomes sufficiently small at larger *W*_P_ values, and that resistance components independent of *W*_P_, such as electrodes, interconnects, and current paths within the paper substrate, increasingly govern the total internal resistance.

Figure 5d shows the dependence of *V*_OC_. For all geometries and *W*_P_ values, *V*_OC_ exhibits a clear linear relationship with Δ*T*, confirming stable thermoelectric behavior. Within the same Δ*T* range, the variation in *V*_OC_ among different *W*_P_ values remains within ± 10–15%, indicating that the influence of *W*_P_ on *V*_*OC*_ is small and that *V*_OC_ is primarily governed by *n*_T_ and Δ*T*. Figure 5e and f show *P*, as a function of *I*_L_ for the Planar and Rolled TEGs at a hot plate set temperature of 100 °C. In both geometries, the *P*–*I*_L_ curves are parabolic and reach a maximum at the vertex. In the Planar TEG, increasing *W*_P_ shifts the vertex toward higher current, and the maximum output is obtained at *W*_P_ = 5.0 mm, indicating saturation of the leg-width effect. In the Rolled TEG, *W*_P_ = 1.5 mm results in the lowest output, whereas the maximum appears at *W*_P_ = 5.0 mm as same as the Planar TEG. When comparing the two geometries at the same *W*_P_, the vertex for the Rolled TEG is consistently lower and shifted toward lower current, indicating that the maximum output decreases after transformation from the Planar to the Rolled geometry for all *W*_P_ values.

Figure 5g shows how *P*_max_ varies with Δ*T* for each *W*_P_. Overall, *P*_max_ tends to be higher for the Planar TEG than for the Rolled TEG within the examined ranges. The reduction in *P*_max_ after rolling remains moderate, indicating that transformation into the Rolled geometry does not severely degrade the power output. Within the Rolled TEG, *P*_max_ increases as *W*_P_ is increased from 1.5 mm to an intermediate range and then decreases at larger *W*_P_. At a hot plate set temperature of 100 °C, the highest *P*_max_ is observed at *W*_P_ = 5.0 mm. This behavior indicates saturation of the output enhancement associated with leg widening, consistent with the saturation trend observed in the internal resistance. Figure 5h shows the Δ*T* dependence of *P*_max,d_. Within the measured Δ*T* range and the *W*_P_ values examined, *P*_max,d_ is consistently higher for the Rolled TEG than for the Planar TEG, reflecting enhanced power generation capability per projected installation area. For example, at a hot plate set temperature of 100 °C and *W*_P_ = 3.0 mm, *P*_max,d_ increases from 0.672 nW cm^−2^ for the Planar TEG to 18.9 nW cm^−2^ for the Rolled TEG, corresponding to an approximately 28.1-fold enhancement. Taken together, these results show that the observed device-level performance reflects both geometry and printed film condition. The effect of *n*_P_ includes changes in PEDOT:PSS film coverage and conduction paths, whereas the effect of *W*_P_ is mainly related to the leg width and conduction cross-section. The nearly unchanged *V*_OC_–Δ*T* slope with varying *W*_P_ indicates that *W*_P_ primarily affects *R*_i_ rather than the device-level Seebeck response. Although intrinsic film properties, such as film-level *S*, *σ*, and *S*^2^*σ*, were not independently measured in this study, the device-level Seebeck coefficients calculated from *V*_OC_/Δ*T* are summarized in Supplementary Table S1. This device-level analysis clarifies how geometry and process-induced film condition govern *R*_i_, *V*_OC_, *P*_max_, and *P*_max,d_. Further film-level characterization will be required to fully decouple material properties from device geometry.

### Scaling the thermocouple number for higher power density

**Fig. 6:**
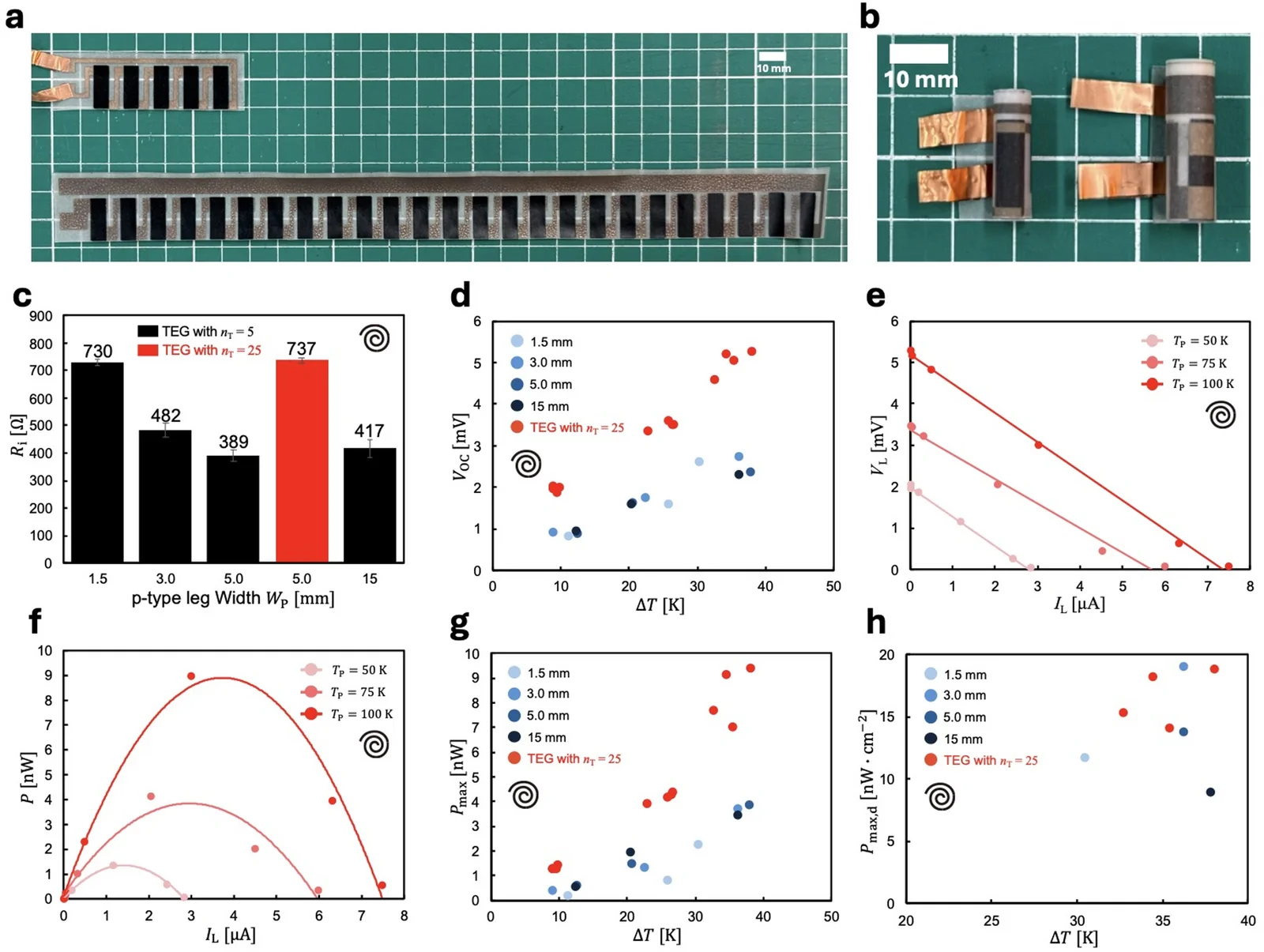
Device structure and output characteristics of the Rolled TEG with increased *n*_T_. (**a**) Photographs of Planar TEGs with *W*_P_ = 5.0 mm and *n*_T_ = 5 and 25, where the thermocouple array extends along the paper substrate as *n*_T_ increases. (**b**) Photographs of Rolled TEGs under the same conditions, where both devices show a similar roll diameter after self-folding. (**c**) Comparison of *R*_i_ between the Rolled TEG with *n*_T_ = 5 and *n*_T_ = 25, where widening the AgNP interconnects suppresses the increase in *R*_i_ to less than twofold even though *n*_T_ is increased fivefold. (**d**) Δ*T* dependence of *V*_OC_. All devices show a clear linear relationship between *V*_OC_ and Δ*T*, and the Rolled TEG with *n*_T_ = 25 shows higher *V*_OC_. (**e**) *V–I* characteristics. Voltage and current show a linear relationship. (**f**) *P–I* characteristics. Output power shows a parabolic curve with a single peak for each temperature condition, and the entire curve shifts toward higher power and higher current as Δ*T* increases. (**g**) Δ*T* dependence of *P*_max_. The Rolled TEG with *n*_T_ = 25 shows a higher *P*_max_, reaching a 2.75-fold increase at Δ*T* = 38 K. (**h**) Δ*T* dependence of *P*_max,d_. The Rolled TEG with *n*_T_ = 25 shows a higher *P*_max,d_, reaching a 1.37-fold increase at Δ*T* = 38 K.

In this section, we investigate how scaling the thermocouple number, *n*_T_, affects the output characteristics and power density of the Rolled TEG. Based on the design conditions identified in the previous sections, we fabricated an improved Rolled TEG with *W*_P_ = 5.0 mm and *n*_T_ increased to 25. The effects of increasing *n*_T_ on *R*_i_, *V*_OC_, *P*_max_, and *P*_max,d_ are evaluated. Figure 6a shows the Planar TEGs with *W*_P_ = 5.0 mm and *n*_T_ = 5 and 25, and Fig. 6b shows the corresponding Rolled TEGs. In the Planar geometry, increasing *n*_T_ extends the thermocouple array along the paper substrate, resulting in a larger in-plane device size. In contrast, in the Rolled geometry, both *n*_T_ = 5 and *n*_T_ = 25 devices exhibit similar outer dimensions after shape transformation, despite the large difference in thermocouple number. This demonstrates that the rolled structure enables a higher thermocouple density without a proportional increase in external dimensions. Table 3 summarizes the design parameters of the devices compared in Fig. 6.

**Table 3:** Design parameters of Rolled TEGs with different *n*_T_.

Type	Electrode length *L* [mm]	p-type electrode width *W*_p_ [mm]	Number of thermocouple *n*_T_	Number of printed PEDOT:PSS layers *n*_P_
1.5	15	1.5	5	10
3.0	15	3.0	5	10
5.0	15	5.0	5	10
15	15	15	5	10
Improved TEG	15	5.0	25	10

Figure 6c compares *R*_i_ for each device. The Rolled TEG evaluated in the previous section, with *W*_P_ = 5.0 mm and *n*_T_ = 5, showed *R*_i_ = 389 Ω, whereas the Rolled TEG fabricated in this section, with *W*_P_ = 5.0 mm and *n*_T_ = 25, showed *R*_i_ = 737 Ω, which corresponds to an increase of 89.5%. When the leg resistance increased strictly in proportion to *n*_T_, a fivefold increase would be expected to result in a much larger increase in *R*_i_. In the present device, the conductive path of the AgNP electrodes on the upper side of the TEG was widened to reduce the series resistance of the electrodes. This design modification compensates part of the resistance increase associated with larger *n*_T_. As a result, the increase in *R*_i_ is effectively suppressed even with a substantial increase in thermocouple number.

Figure 6d shows the Δ*T* dependence of *V*_OC_. For all devices, *V*_OC_ shows a clear linear relationship with Δ*T*, which indicates that a stable thermoelectric response is maintained even in the rolled geometry. The Rolled TEG with *n*_T_ = 25 shows higher *V*_OC_ across the entire Δ*T* range than all the Rolled TEGs with *n*_T_ = 5. For example, at Δ*T* = 37.9 K under the 100 °C hot plate condition, the Rolled TEG with *n*_T_ = 5 shows *V*_OC_ = 1.92 mV, whereas the Rolled TEG with *n*_T_ = 25 shows *V*_OC_ = 3.93 mV, which is a 2.05-fold increase. These results confirm that scaling *n*_T_ effectively enhances *V*_OC_ in the Rolled TEG. Figure 6e shows the voltage current characteristics of the Rolled TEG with *n*_T_ = 25 measured at hot plate set temperatures of 50, 75, and 100 °C. Linear voltage–current relations are observed under all temperature conditions, indicating ohmic behavior and stable electrical characteristics in the rolled geometry.

Figure 6f shows the power-current characteristics. With increasing Δ*T*, the entire parabola shifts toward higher power, and the peak moves toward higher power and higher current. Figure 6g compares the Δ*T* dependence of *P*_max_. The Rolled TEG with *n*_T_ = 25 shows higher *P*_max_ than the Rolled TEG with *n*_T_ = 5, which indicates that increasing *n*_T_ is effective for improving *P*_max_ in the Rolled TEG. As a representative case at a hot plate temperature of 100 °C, the Rolled TEG with *n*_T_ = 25 shows *P*_max_ = 9.42 nW at Δ*T* = 38.1 K, whereas the Rolled TEG with *n*_T_ = 5 shows *P*_max_ = 3.43 nW at a comparable Δ*T* = 37.9 K, which corresponds to a 2.75-fold increase in *P*_max_.

Figure 6h shows the Δ*T* dependence of *P*_max,d_. The Rolled TEG with *n*_T_ = 25 shows higher *P*_max,d_, which indicates that increasing *n*_T_ is also effective for improving footprint normalized power generation capability. As a representative example under the 100 °C hot plate condition, the Rolled TEG with *n*_T_ = 25 shows *P*_max,d_ = 18.7 nW cm^−2^ at Δ*T* = 38.1 K, whereas the Rolled TEG with *n*_T_ = 5 shows *P*_max,d_ = 13.7 nW cm^-2^ at Δ*T* = 36.3 K, which corresponds to a 1.37-fold increase. These results indicate that increasing *n*_T_ while maintaining nearly the same projected footprint of the rolled structure enhances both the output and the power density normalized by the installation area.

### Demonstration of temperature sensing using the rolled TEG

In this section, we evaluate the feasibility of the proposed Rolled TEG as a paper based environmental sensor by leveraging its thermoelectric voltage generation characteristics. As the sensing element, we used the Double Couple Rolled TEG, which showed the highest open circuit voltage. Using a device with a higher output voltage is important for ensuring the signal to noise ratio, SNR, which enables more accurate temperature measurements. In addition, because a thermoelectric device can generate a voltage without an external power source, the proposed Rolled TEG suggests a future path toward battery-less sensors that obtain signals using only ambient energy harvesting.

In this demonstration, we treated the Rolled TEG as a temperature dependent voltage source, and we built a measurement system that estimates temperature from its output. Specifically, we prepared two calibration approaches for the voltage temperature relationship. In Case 1, we first converted the voltage into a temperature difference, then converted the temperature difference into temperature. In Case 2, we directly converted the voltage into temperature. We compared these approaches in terms of temperature estimation accuracy and implementation simplicity.

**Fig. 7:**
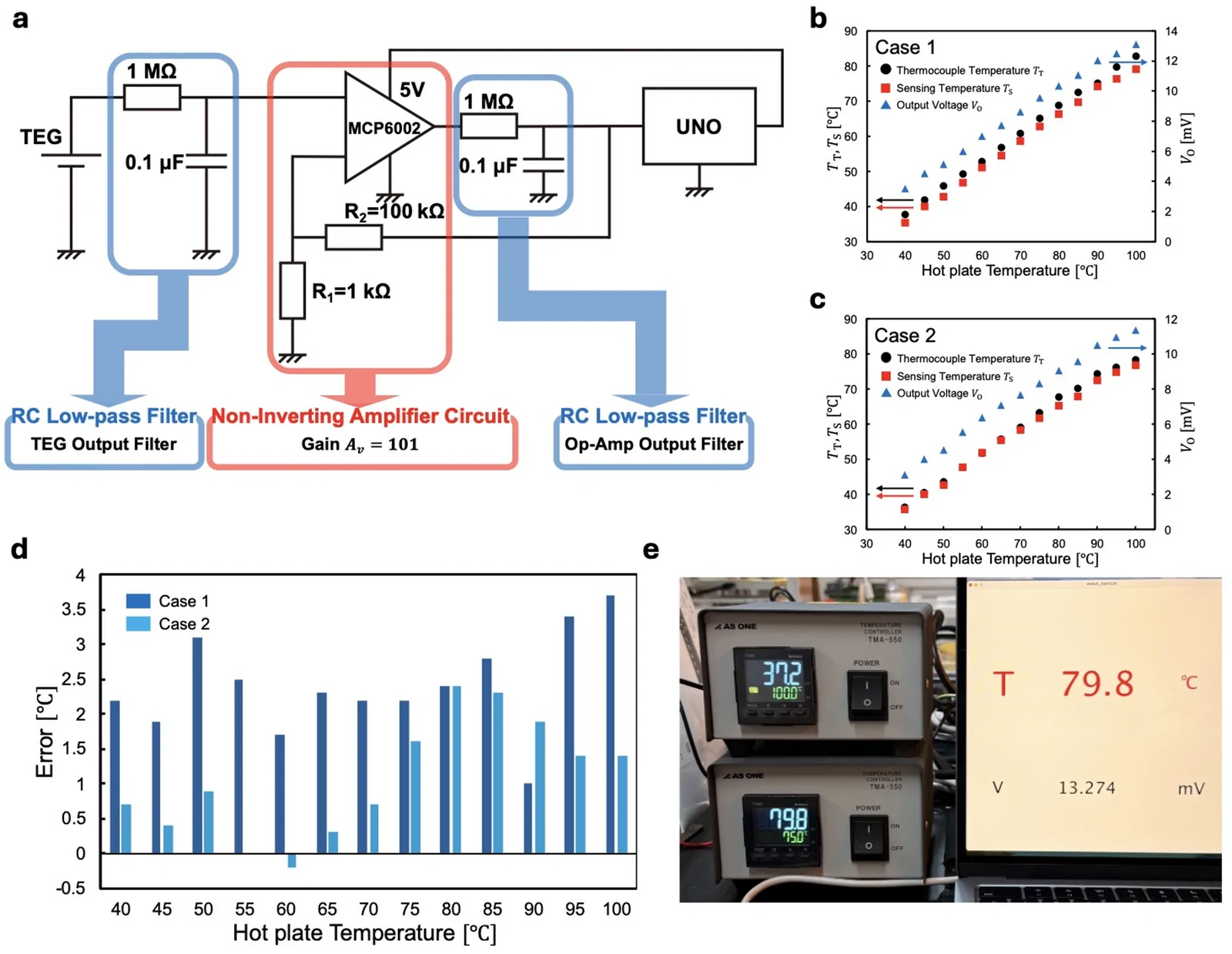
Demonstration of temperature sensing using the Rolled TEG. (**a**) Schematic of the temperature sensing circuit using a Double Couple Rolled TEG. The output voltage of the Rolled TEG was conditioned and digitized using an analog front-end and a microcontroller, and then converted into temperature. (**b**) Results for Case 1, the sensing temperature *T*_S_ was obtained from the output voltage *V*_O_ via the temperature difference Δ*T*, then compared with the thermocouple temperature *T*_T_. (**c**) Results for Case 2, *T*_S_ was directly calculated from *V*_O_ using a linear equation, then compared with *T*_T_. (**d**) Comparison of the difference between *T*_S_ and *T*_T_ for Case 1 and Case 2. Case 2 achieved temperature estimation with smaller error. (**e**) Photograph of the temperature-sensing experiment. The temperature at the hot side was successfully estimated from the voltage measured by the Rolled TEG and displayed on the monitor in real time.

Figure 7a shows an overview of the temperature sensing system developed in this study. The operational amplifier circuit and the microcontroller are powered by an external supply, and the TEG itself operates as a passive sensing element. The TEG output voltage is first smoothed using an RC filter, then amplified to the desired voltage range using a high input impedance, non-inverting amplifier. The signal is then passed through another RC filter to suppress high-frequency noise, and it is fed into the built-in A/D converter of the microcontroller. By applying the calibration equation to the digitized signal, we calculate the hot plate side temperature of the Rolled TEG, namely the sensing temperature, *T*_S_, at the lower end of the Rolled TEG, which is the hot side.

Figure 7b shows the results of the two-step calibration, Case 1, where we first estimate the temperature difference Δ*T* from the output voltage *V*_O_, then estimate the sensing temperature *T*_S_ from Δ*T*. First, we varied the hot plate set temperature in 5 °C increments, and we simultaneously measured *V*_O_ and Δ*T*, which was measured using thermocouples. We fitted a linear model to the relationship between *V*_O_ and Δ*T*, and we obtained,

3 $$\Delta T=3.17\cdot {V}_{\mathrm{O}}+0.528$$

Next, we approximated the relationship between the hot side temperature, the sensing temperature *T*_S_, and Δ*T* under the same conditions using a first order model, and we obtained,

4 $${T}_{\mathrm{S}}=1.52\cdot \Delta T+20.7$$

By substituting Eq. (3) into Eq. (4), we finally derived a linear relationship between *V*_O_ and *T*_S_,

5 $${T}_{\mathrm{S}}=4.98\cdot {V}_{\mathrm{O}}+21.6$$

These results confirm that the hot side temperature of the Rolled TEG can be calculated from *V*_O_ via the two-step conversion, *V*_O_ to Δ*T* to *T*_S_.

Figure 7c shows the results of the one-step calibration, Case 2, where *T*_S_ is directly estimated from *V*_O_. Here, we varied the hot plate set temperature in the same 5 °C increments, and we simultaneously measured *V*_O_ and *T*_S_. We fitted a first order model to their relationship, and we obtained,

6 $${T}_{\mathrm{S}}=4.99\cdot {V}_{\mathrm{O}}+20.0$$

The slope and intercept of Eq. (5) and Eq. (6) agree well with each other, which indicates a clear linear relationship between *V*_O_ and Ts for the Rolled TEG. Therefore, for the Rolled TEG in this study, *T*_S_ can be directly converted from *V*_O_ using a single linear equation, without explicitly estimating Δ*T*.

Figure 7d summarizes the temperature difference between *T*_S_ calculated from the Rolled TEG output voltage and *T*_T_ measured by a thermocouple placed the same location for Case 1 and Case 2. In both methods, *T*s follows *T*_T_ well, and the average error ranges were ± 2.42 °C for Case 1 and ± 1.03 °C for Case 2. The improved temperature estimation accuracy is attributed to the increased thermocouple number *n*_T_, which enhances the output voltage amplitude for a given temperature difference and thereby improves the voltage-to-temperature resolution. Under our measurement conditions, the direct conversion from voltage to temperature, Case 2, enables temperature estimation with smaller error. In addition, Case 2 is expressed by a single linear equation, which simplifies the implementation on the microcontroller and is advantageous in terms of computational load.

Figure 7e shows the temperature sensing demonstration. The Rolled TEG was placed on a hot plate, and a thermocouple was attached to the hot plate surface near the device position to measure *T*_T_ as the reference temperature. On the right monitor, the microcontroller reads the Rolled TEG output voltage *V*_O_ and displays *T*_S_ in real time, converted based on Eq. (6). This real-time display allows direct evaluation of temperature estimation using the Rolled TEG by comparing the temperature derived from the TEG output with the thermocouple-based reference. Overall, this temperature sensing experiment is positioned as a proof-of-concept demonstration of the Rolled TEG as a thermoelectric sensing element, rather than as a comprehensive replacement for conventional temperature sensors. The voltage-temperature relationships shown in Fig. 7b and c were fitted with linear functions, and their slopes were used to define the voltage-to-temperature calibration sensitivity of the temperature sensor based on the Rolled TEG. The linearity was evaluated using the coefficient of determination, *R*^2^ = 0.9976 for Case 1 and *R*^2^ = 0.9957 for Case 2, indicating high linearity in the voltage-temperature calibration. The temperature sensing range examined in this study was 40 to 100 °C, corresponding to the hot plate temperature range used for calibration and demonstration. Response and recovery times were not determined in this study because their quantitative evaluation requires dedicated temperature step experiments. The practical upper operating temperature should be further validated because it can be affected by the thermal durability of the paper substrate and printed PEDOT:PSS/AgNP patterns. In addition, the effects of environmental variations, such as relative humidity, and external mechanical deformation, such as bending or pressing, on the temperature sensing performance were not systematically evaluated in this study. Because these factors can affect the paper substrate, printed PEDOT:PSS/AgNP patterns, electrical contacts, and thermal boundary conditions, dedicated environmental and mechanical reliability tests will be required in future work. The long-term electrical stability was evaluated by measuring V_OC_ and R_i_ over 14 days using three independently fabricated Planar TEGs and three independently fabricated Rolled TEGs, as shown in Supplementary Figs. S2 and S3. The Rolled TEG maintained stable V_OC_ at hot plate set temperatures of 50, 75, and 100 °C, and Ri showed only a small change during the measurement period. These results indicate that the basic thermoelectric response and electrical continuity of the Rolled TEG were preserved over 14 days. In addition, the material cost was estimated from the amounts and unit prices of AgNP ink, PEDOT:PSS, and the paper substrate. The estimated material cost of a representative Rolled TEG with *W*_P_ = 5.0 mm in Fig. 5 was 14.67 USD per device.

## Conclusion

In this study, we demonstrated a rolled organic thermoelectric generator that autonomously transforms from a planar device into a rolled geometry by paper self-folding combined with a fully printed fabrication process. We systematically compared its power generation characteristics with those of the planar geometry. Despite the large structural transformation, the rolled device preserved a linear Seebeck response and ohmic behavior, confirming that it functions as a thermoelectric generator after rolling. Increasing *n*_P_ substantially reduced the internal resistance, and in the rolled geometry, widening *W*_P_ was effective up to *W*_P_ = 5.0 mm, beyond which the resistance reduction saturated. At a hot plate temperature of 100 °C, the rolled TEG achieved a maximum power density of 18.9 nW cm^−2^ at *W*_P_ = 3.0 mm, which is 28.1 times higher than that of the planar TEG. This increase in power density is mainly attributed to the compact rearrangement of the thermocouple array within a smaller projected footprint, rather than to a large increase in the absolute maximum output power. Moreover, increasing the thermocouple number from *n*_T_ = 5 to 25 while maintaining a nearly unchanged projected footprint enabled a 2.75-fold increase in maximum power and a 1.37-fold increase in footprint-normalized power density. In addition to power generation, the rolled TEG was demonstrated as a temperature sensor, where the output voltage showed good agreement with reference thermocouple measurements with an error within ± 1.03 °C, indicating that accurate temperature sensing is preserved after rolling. This result should be regarded as a proof-of-concept demonstration of the Rolled TEG as a thermoelectric sensing element. The advantage of this approach is not to replace conventional temperature sensors in terms of general sensing accuracy, but to generate a temperature-difference-dependent voltage from the TEG itself without an external power supply for the sensing element. In the present demonstration, an external circuit was used to amplify and read out the voltage signal. Future integration with low-power signal processing and communication circuits may enable battery-less temperature sensing systems in space-constrained applications, such as distributed IoT nodes and compact sensing modules. These results establish paper self-folding as an effective and scalable strategy for achieving three-dimensional densification of fully printed organic TEGs, enabling substantial improvements in footprint-normalized device-level power density through compact three-dimensional integration without increasing planar area, and providing a promising platform for distributed, lightweight, and environmentally benign energy and sensing systems. The functional advantage of the rolled geometry is therefore not simply the shape transformation itself, but the compact three-dimensional integration of the printed thermocouple array within a limited projected footprint. This feature is particularly relevant for space-constrained applications, such as distributed IoT nodes and compact sensing modules, where thermoelectric devices must be integrated into a small installation area while retaining power generation and sensing functions.

## Experimental section

### Common measurement conditions

The power generation and electrical characteristics of the fabricated TEGs were evaluated at a room temperature of 21 °C. The internal resistance, *R*_i_, was measured using a digital multimeter (VOAC7522H, Iwasaki Tsushinki Co., Ltd.). For power generation measurements, a hot plate (EC-1200NP, AS ONE) was used as the heat source to apply a temperature difference, Δ*T*, across the device. A variable resistor (DBR-06, Joman Co., Ltd.) was connected to the TEG as an external load resistance, *R*_L_. The load voltage, *V*_L_, and load current, *I*_L_, were measured while sweeping *R*_L_. The output power was calculated as *P* = *V*_L_^2^/*R*_L_, and the maximum power, *P*_max_, was determined from the resulting power characteristics. The open-circuit voltage, *V*_OC_, was measured under open-circuit conditions. All electrical data were recorded using the digital multimeter. The open-circuit voltage, *V*_OC_, was measured under open-circuit conditions. All electrical data were recorded using the digital multimeter. For the *R*_i_ data shown with error bars in Figs. 3c, 4e, 5c, and 6c, the plotted values represent the mean ± standard deviation calculated from repeated *R*_i_ measurements using independently fabricated devices. Specifically, the *R*_i_ values represent the mean of three devices in Fig. 3c, two devices in Fig. 4e, three devices in Fig. 5c, and four Rolled TEG devices with *n*_T_ = 25 in Fig. 6c. Each device was measured at hot plate set temperatures of 50, 75, and 100 °C. The data for the Rolled TEG with *n*_T_ = 5 in Fig. 6c are the same as those shown in Fig. 5c. For the long-term stability measurements shown in Supplementary Figs. S2 and S3, three independently fabricated Planar TEGs and three independently fabricated Rolled TEGs were evaluated on days 0, 1, 3, 7, and 14. Each device was measured three times for each measurement condition on each measurement day, and the plotted values represent the mean ± standard deviation.

### TEG fabrication

The interconnects were formed by inkjet printing a silver nanoparticle (AgNP) ink (DryCure Ag-J 0410B, C-INK Co., Ltd.) on tracing paper as the substrate. After printing, the entire device was thermally treated by drying and sintering on a hot plate at 200 °C in air to obtain conductive interconnects. The p type thermoelectric legs were then formed by screen printing PEDOT:PSS (product no. 768650, Sigma-Aldrich) onto the substrate and drying on a hot plate at 70 °C for 10 min in air. The PEDOT:PSS printing and drying steps were repeated to achieve the desired printing count for the *p* type legs. Inkjet printing was performed using an inkjet printer (PX-S155, Seiko Epson Corporation). For screen printing, the screen was prepared using a 120 mesh (MiScreen a4 screen master, 8316, RISO Kagaku Corporation), a plastic frame (MiScreen a4 plastic frame, 8315, RISO Kagaku Corporation), and a digital screen maker (MiScreen a4, 7767, RISO Kagaku Corporation), and printing was performed using a urethane squeegee (100 × 120 mm, Otsuka Brush Mfg. Co., Ltd.). The self-folding based printing process followed the procedure reported in our previous studies ^26,27^.

### Planar TEG measurement

The Planar TEG was positioned to bridge the hot plate and an aluminum heat sink. By bringing one end of the legs into contact with the hot plate surface and the other end into contact with the heat sink surface, a temperature gradient was formed along the leg length.

### Rolled TEG measurement

The Rolled TEG was placed upright on the hot plate. To stabilize thermal contact during measurement, and to prevent tipping or posture changes, a paper cup fixture containing a small weight was placed over the top of the TEG. Temperatures were monitored using K-type thermocouples (TMA-550K, AS ONE) at the lower end of the Rolled TEG, which served as the hot side, and at the upper end, which served as the cold side, then their difference was defined as Δ*T*.

### Leg surface observation

The surface morphology and roughness of the PEDOT:PSS legs were observed and measured using a laser microscope (OLS5100, Shimadzu Corporation). To evaluate how the number of printed PEDOT:PSS layers, *n*_P_, affects the surface state, surface images were acquired for both pre-folding and post-folding samples. As surface roughness metrics, the arithmetic mean height, *S*_*a*_, and the root mean square height, *S*_*q*_, were calculated within a selected measurement area.

### Temperature sensing demonstration setup

For the temperature sensing demonstration, the TEG output voltage was fed into a non-inverting amplifier circuit using an operational amplifier (MCP6002-E/P, Microchip Technology Inc.), and the amplified signal was acquired by the A/D converter of a microcontroller board (Arduino Uno).

## Supplementary Information

Below is the link to the electronic supplementary material.

Supplementary Material 1

## Data Availability

The datasets generated and/or analysed during the current study are not publicly available because they are part of an ongoing study and further analyses are planned, but are available from the corresponding author on reasonable request.
