# Supplementary material for: High power density in a fully printed origami-inspired rolled thermoelectric generator enabled by paper self-folding
Source: Sci Rep. 2026 Jun 17;16:27749. doi: 10.1038/s41598-026-57024-0 (PMC13542274; doi:10.1038/s41598-026-57024-0)
Supplement: Supplementary file 1 [file 41598_2026_57024_MOESM1_ESM.docx]

**High Power Density in a Fully Printed Origami-Inspired Rolled Thermoelectric Generator Enabled by Paper Self-Folding**

*Hayato Yokoyama*^1^, *Motoki Maeda*^1^, *Giovanna Latronico*^2^, *Paolo Mele*^3^, *Hiroki Shigemune*^3^

1. *Graduate School of Engineering Science*

*Shibaura Institute of Technology*

*3-7-5 Toyosu, Koto-ku, Tokyo 134-8548, Japan*

2. *CNR-ICMATE*

*Institute of Condensed Matter Chemistry and Technologies for Energy, National Research Council, Via Previati 1/E, 23900 Lecco, Italy*

3. *College of Engineering*

*Shibaura Institute of Technology*

*3-7-5 Toyosu, Koto-ku, Tokyo 134-8548, Japan*

** Corresponding author Email: hshige@shibaura-it.ac.jp*

Table S1. Comparison of device structures and output characteristics for the different TEG conditions investigated in this study.

Table S2. Comparison of device structure, fabrication methods, and output characteristics of this work and related TEGs.

38. Brunetti, I. et al. A Scalable Fully Printed Organic Thermoelectric Generator for Harsh Environments Enabled by a Stable n-type Polymer. *Adv. Mater. Technol.* **10**, 2400968 (2025).
39. Pataki, N. J. et al. A Label-Like Monolithic Organic Thermoelectric Generator Enabled by Local Inkjet Doping of Aligned Polymer Films. *Adv. Energy Mater.* **15**, 2404656 (2025).

**Figure S1 | Sample-to-sample variation in *P*–*I*_L_ characteristics of Rolled TEGs with *n*_T_ = 25.** *P*–*I*_L_ characteristics of four independently fabricated Rolled TEGs with *n*_T_ = 25 measured at hot plate set temperatures of (a) 50 °C, (b) 75 °C, and (c) 100 °C. The data reported in the main text are shown together with the other sample data. The *P*–*I*_L_ curves show only small sample-to-sample variation at each hot plate set temperature, confirming the reproducibility of the output characteristics.

**Figure. S2 | Long-term stability of open-circuit voltage, *V_OC_*, over 14 days.** Changes in *V_OC_* of the Planar and Rolled TEGs measured at hot plate set temperatures of (a) 50 °C, (b) 75 °C, and (c) 100 °C. Measurements were performed on days 0, 1, 3, 7, and 14. The Rolled TEG maintained stable *V_OC_* at each hot plate set temperature, indicating that the basic thermoelectric response was preserved over 14 days.

**Figure. S3 | Long-term stability of internal resistance, *R_i_*, over 14 days.** Changes in *R_i_* of the Planar and Rolled TEGs measured on days 0, 1, 3, 7, and 14. *R_i_* showed only a small change during the measurement period, indicating that electrical continuity was preserved over 14 days.
